# Management of hair loss associated with endocrine therapy in patients with breast cancer: an overview

**DOI:** 10.1186/s40064-016-2216-3

**Published:** 2016-05-10

**Authors:** Fatih Karatas, Suleyman Sahin, Ali R. Sever, Kadri Altundag

**Affiliations:** Department of Medical Oncology, Diskapi Yildirim Beyazit Education and Training Hospital, Ankara, Turkey; Department of Radiology, Hacettepe University School of Medicine, Ankara, Turkey; Department of Medical Oncology, Hacettepe University Cancer Institute, 06100 Ankara, Turkey

**Keywords:** Endocrine therapy, Breast cancer, Hair loss, Management

## Abstract

Endocrine therapy-induced hair loss (ETIHL) associated with aromatase inhibitors and tamoxifen treatment is currently mostly reported but remained an unresolved therapeutic issue in patients with breast cancer (BC) since the number of studies regarding the management is limited in literature. Herein we investigated the possible causes of this clinical problem and its relation with endocrine therapies widely used for BC survivors and made some modest practical recommendations in light of the literature review in order to provide an optimal management. On the basis of literature findings, common causes of hair loss apart from endocrine therapies should be investigated with an initial evaluation workup and then should be corrected, if observed. Treatment with topical 5-alpha reductase inhibitors and supplementation of Vitamin C and omega-3 fatty acids are likely appeared to be the most appropriate treatment agents for ETIHL without causing an adverse effect on BC prognosis. However, more prospective, randomised, placebo-controlled studied are required in order to confirm our results and also identify the clinical effects of this problem on patients with BC.

## Background

Tamoxifen and Aromatase Inhibitors (AIs) currently hold an important place as endocrine therapy (ET) in adjuvant and/or metastatic setting of the patients with Hormone Receptor (i.e. Estrogen Receptor+ and/or Progesterone Receptor+) positive invasive or non-invasive breast cancer (BC) (Early Breast Cancer Trialists’ Collaborative Group (EBCTCG) 2005; Howell et al. 2005). However, a substantial portion of the patients using tamoxifen therapy for many years are reported to suffer from the hair loss (HL) or hair thinning (Gallicchio et al. 2013). A retrospective questioning of the patients with BC regarding this therapeutic issue has revealed that approximately 25 % of the patients receiving ET experience HL or thinning (Gallicchio et al. 2013). So that, this rate is almost similar to those reported for flushing and arthralgia related to endocrine therapy which are well-known to impair the quality of life and more considered by physicians for specific additional medications (Niravath 2013; Laroche et al. 2014). Prolonged exposure to androgenic effects that is increased with aging in men leads to frontoparietal baldness defined as “male pattern baldness”, however, HL in women arises on the midscalp and/or globalscalp due to the diminished estrogen levels in advanced ages (Olsen 2001). Hair texture of the scalp consists a plenty of estrogen and androgen receptors, particularly frontal and parietal area (Wood and Price 1999). An assessment of androgenetic alopecia with a comparison of 12 women (aged 14–33 years) and 12 men (aged 18–30 years) showed that frontal scalp had more androgen and 5-alpha reductase receptor in comparison to occipital scalp in both sexes. However, relative values were found to be much more greater in men compared to women (Sawaya and Price 1997). This explains the difference patterns of HL in men and women in normal circumstances. AIs widely used in BC treatment (letrozole, anastrozole, exemestan) and tamoxifen known as selective estrogen receptor modulator are known to cause HL (male pattern baldness) or hair thinning in women (Gallicchio et al. 2013). Rebound increased levels of androgen alongside the diminished levels of estrogen due to AIs are supposed to decelerate and inhibit the proliferation of the hair follicles in the skin (Rossi et al. 2013). Reduced estrogenic effects due to tamoxifen enables the hair folliclesis go into the resting phase (Gateley and Bundred 1997). These mechanisms mentioned above may be the main reasons of HL caused by ET. Besides, one another possible mechanism may be explained with the theory of that tamoxifen or AIs lead to HL directly on growth receptors or through immunological mechanisms, however, a study with respect to this issue has not yet been performed.

Although it is known that chemotherapy-induced HL during adjuvant chemotherapy is a temporary clinical issue, it constitutes a great concern and cosmetic problem in patients with BC. Many patients therefore tries to overcome this period by covering their heads with a wig, however, this situation may psychologically leads to treatment rejection or discontinuation. By contrast, although patients with advanced disease treated with chemotherapy also faced with similar HL problems, cosmetic care in such patients generally remains in the background as they are primarily aware of the importance of the metastatic process along with the treatment response (Lemieux et al. 2008). Thus, because the endocrine therapy-induced hair loss (ETIHL) negatively effects the sociocultural status and quality of life, it is a major problem and remains a therapeutic challenge in patients with BC, hence should be managed by effective treatment options which are capable to stop the HL rather than additional psychological or prosthetic options.

## Current recommendations for chemotherapy induced hair loss

Minoxidil is a vasodilator drug, it is considered a drug to promote hair growth (Olsen et al. 1985). So far, numerous of agents and methods such as cold application to scalp or local minoxidil therapy have been used during chemotherapy period for the management of HL that negatively effects the quality of life among BC survivors. While minoxidil has been shown to provide a faster hair growth rather than preventing the HL in clinical trails (Wang et al. 2006), despite a positive treatment effect, cold application to the scalp has not been considered to be effective and useful in this direction since it causes lots of complications such as infection and thrombosis (Trüeb 2010). Although AIs or tamoxifen-induced HL is a common clinical entity and problem for both oncologists and their patients and also frequently asked and encountered clinical issue which leads patients to seek for a remedy at websites (Tamoxifen and March 2016), it is surprising and distressing that no randomized controlled studies with respect to this issue have yet been designed. Theoretically, the direct implementation of the estrogenic effective creams on the scalp tissue may decrease the ETIHL. However, when considering the AI therapy to be used for a total of 5 years, whether the systemic transition risk of the estrogenic therapy plays a negative effect on disease recurrence in patients with BC is still an enigma and also requires to be clarified in light of the randomized, placebo-controlled trials to demonstrate the efficacy and safety of this treatment. Herein, we have evaluated the possible role of most recently suggested nutritional supplements and 5-a reductase inhibitors with considering the possible hormonal mechanisms in the prevention of ETIHL.

## Possible treatment options for ETIHL

For the purpose of preventing the ETIHL, reducing the rebound androgenic effects by maintaining the anti-estrogenic effect may theoretically act as a potential beneficial method for the hair follicle. To do so, combining the endocrine therapies (such as LHRH agonists) with mitotane and ketoconazole which are used to disrupt the ovarian or adrenal androgenic hormones synthesis may provide this effect (Santen and Samojlik 2013). However, some suppression therapies of adrenal glands functions, particularly ketoconazole, have various side-effects such as adrenal insufficiency and hepatic enzyme suppression (Boetsch et al. 2015).

In healthy adults, estrogen is known to play a key role in the hair development in tissue levels and also increase the amino acids and trace elements (such as selenium, cysteine) in hair texture (Zhou et al. 2012). For this reason, trace elements and/or vitamin supplements may prevent the ETIHL. Moreover, one another important example to demonstrate the beneficial role of vitamins on hair health is that vitamin B6 is known to decrease the efficacy of the estrogen in hair texture or other tissues (Finner 2013; Hertz 1948). Today, even though the opinion of that B6 supplements (topical or systemic) may be used to treat the baldness, this can theoretically reduce the estrogenic effects on the scalp, hence may not be effective in the management of ETIHL.

Vitamin C potentiates the estrogenic effects in vascular levels and also reduces the effectiveness of testosterone (Hwang et al. 2000). Alongside the increased estrogenic activity, vitamin C also has a protective role against the BC (Kim et al. 2006), coronary artery disease (Hwang et al. 2000) and osteoporosis (Sahni et al. 2009). Thus, vitamin C may loco regionally reduces the HL by increased estrogenic effects along with decreased androgenic effects on the scalp, when locally applied with an oil-based buffer that enables vitamin C to pass across the hair texture.

While the low levels of vitamin D are suggested to have an adverse effect on the survival of patients with BC, supplementation of vitamin D deficiency is known to improve the survival (Mohr et al. 2014). Besides, low levels of vitamin D in serum and/or lack of Vitamin D receptor have been found to be associated with HL (Rasheed et al. 2013; Amor et al. 2010). However, it is still uncertain whether vitamin D supplementation increases the proliferation of hair follicles (Amor et al. 2010; Rosen et al. 2012).

Vitamin E has been shown to decrease the effectiveness of estrogen in the breast tissue and low levels of vitamin E are also reported to be associated with increased levels of estrogen (Chamras et al. 2005). However, according to the results of the SELECT study in which an increased rate of prostate cancer was reported, Vitamin E carried a potential androgenic and anti-estrogenic activity in patients receiving Vitamin E supplementation (Dunn et al. 2010).

Folic acid is also reported to have a positive effect on hair health (Rushton 2002), however, unfortunately, there are some epidemiological studies that suggest folate may increase the tissue levels of estrogen, indicating that high levels of folic acid might lead to BC (Kim 2006).

Oral supplementation of omega-3 and -6 fatty acids is also known to positively affect (Pardini 2006) the prognosis of BC (Bartsch et al. 1999) and many other cancers and also increase the apoptosis of BC cells (Sun et al. 2011) in tissue cultures. Additionally, it has been recently shown that 6 months after the use of these fatty acids were found to protect and improve the hair health through antioxidant effects on the scalp tissue (Floc’h et al. 2015).

Apart from nutritional supplements, another important point for the potential treatment of ETIHL is hormonal manipulation, undoubtedly. Because AIs block the formation of estrogen from androgens, more testosterone synthesis may occur from the androgens accumulated behind, hence might increase the 5-alpha reductase enzyme activity in tissue levels, leading to much more dihydrotestosterone formation that is known to be the most potent androgenic structure in human body. Therefore, specific and systemic treatment with anti-5-alpha reductase agents (such as finasteride or dutasteride) may preclude both HL on the scalp and male pattern hair growth in other parts of the body. Genuinely, the fact that patients with deficiency of congenital 5-alpha reductase enzyme activity rarely present (Herskovitz and Tosti 2013) with male pattern HL or that patients using a 5-alpha reductase enzyme inhibitors have an significant improvement (Olsen et al. 2006) in HL is the objective evidence of this situation. By contrast, given the systemic side effects of these agents, topical 5-alpha reductase enzyme inhibitors may be locally (particularly frontal-temporal regions) or entirely applied on the scalp skin.

## Rationality for safety and availability

When taking into account the aforementioned potential positive effects and risks of the treatment strategies, vitamin C and folic acid, providing only local use, are more likely to be the most potential and effective treatment agents due to the fact that they have a short half-life duration and that vitamin C is stored in the body for a long time, leading to increased levels in the systemic circulation and preventing the breast cancer recurrence. Moreover, vitamin C may have a protective role from AI-induced osteoporosis, providing a more potentiate effect to the current osteoporosis treatments. Because to positive direction of hair follicle proliferation stimulating effect of the vitamin D receptor is ligand independent, Vitamin D supplementation should not be much of a contribution on ETIHL. Besides, omega-3 fatty acids are another potential treatment agents in the prevention of ETIHL because of having positive effects on both breast tissue and hair health. Based upon these findings, when considering the underlying mechanism, we emphasize that 5-alpha reductase enzyme inhibitors (local cream or shampoo forms) could possibly be the most appropriate and potent treatment options in the treatment of ETIHL. It is also clear that systemic suppression methods of androgen synthesis may not be appropriate options due to systemic potential side effects (for instance; adrenal insufficiency)which can not be worth to take risk for cosmetic purposes and may also lead to potential comorbidities or mortalities. Despite having no adverse effect on BC prognosis, vitamin E does not appear to be beneficial since they might provoke the male pattern HL by its possible androgenic effects.

## Summary

Endocrine therapy-induced HL have remained an important but less cared cosmetic problem in patients with breast cancer. When considering that one forth of the patients with breast cancer using endocrine therapy encounter with this clinical problem; rather than suggesting patients anti-HL methods, it would be more beneficial and effective to suggest them (before they start the complaining of HL problem) to be aware of that HL could be resolved. Today, there is no evidence based therapy with regard to this issue. In addition to those suggestions, before planning an initial endocrine therapy, several factors which may lead to HL including zinc and trace element deficiencies, other endocrine disorders and fungal or bacterial infections on the scalp should be primarily evaluated in patients suffering ETIHL.

In conclusion, we would like to emphasise that topical 5-alpha reductase inhibitors and supplementation of vitamin C and omega 3 fatty acids in a non-toxic dose without leading to any adverse effect on the prognosis of the patients with breast cancer appear to be the most appropriate options in the management. However, more prospective, placebo-controlled studies are currently required to prove the efficacy of these treatment methods in endocrine therapy induced hair loss.
